# The Synthesis of Ascorbic Acid in Rice Roots Plays an Important Role in the Salt Tolerance of Rice by Scavenging ROS

**DOI:** 10.3390/ijms19113347

**Published:** 2018-10-26

**Authors:** Yayun Wang, Hui Zhao, Hua Qin, Zixuan Li, Hai Liu, Juan Wang, Haiwen Zhang, Ruidang Quan, Rongfeng Huang, Zhijin Zhang

**Affiliations:** 1 Biotechnology Research Institute, Chinese Academy of Agricultural Sciences, Beijing 100081, China; wangyayun13@163.com (Y.W.); zhaohui_hbu@126.com (H.Z.); qinghua812@yeah.net (H.Q.); Lizixuan0707@163.com (Z.L.); wangjuan@caas.cn (J.W.); zhanghaiwen@caas.cn (H.Z.); quanruidang@caas.cn (R.Q.); rfhuang@caas.cn (R.H.); 2 National Key Facility of Crop Gene Resources and Genetic Improvement, Beijing 100081, China; 3 Department of Biology, University of Virginia, Charlottesville, VA 22903, USA; hl9h@eservices.virginia.edu

**Keywords:** ascorbic acid, rice, root, salt stress, reactive oxygen species

## Abstract

The root plays an important role in the responses of plants to stresses, but the detailed mechanisms of roots in stress responses are still obscure. The GDP-mannose pyrophosphate synthetase (GMPase) *OsVTC1-3* is a key factor of ascorbic acid (AsA) synthesis in rice roots. The present study showed that the transcript of *OsVTC1-3* was induced by salt stress in roots, but not in leaves. Inhibiting the expression of *OsVTC1-3* by RNA interfering (RI) technology significantly impaired the tolerance of rice to salt stress. The roots of *OsVTC1-3* RI plants rapidly produced more O_2_^−^, and later accumulated amounts of H_2_O_2_ under salt stress, indicating the impaired tolerance of *OsVTC1-3* RI plants to salt stress due to the decreasing ability of scavenging reactive oxygen species (ROS). Moreover, exogenous AsA restored the salt tolerance of *OsVTC1-3* RI plants, indicating that the AsA synthesis in rice roots is an important factor for the response of rice to salt stress. Further studies showed that the salt-induced AsA synthesis was limited in the roots of *OsVTC1-3* RI plants. The above results showed that specifically regulating AsA synthesis to scavenge ROS in rice roots was one of important factors in enhancing the tolerance of rice to salt stress.

## 1. Introduction

Ascorbic acid (known as Vitamin C, AsA), one of the essential vitamins for human beings, also has important roles in growth and development [1,2]. As the most abundant antioxidant of plants, AsA is involved widely in scavenging reactive oxygen species (ROS) during the photosynthetic electron transfer and stress responses [2]. Therefore, the synthesis of AsA is related closely to environmental stresses [2,3].

AsA has been known for about one century [4]. Several alternative pathways, such as galacturonate and glucuronate, etc., have been suggested, and studies showed that the D-mannose/L-galactose pathway is the domain pathway of AsA synthesis in higher plants; all the genes and enzymes involved have been identified [5,6,7,8,9]. The guanosine diphosphate -mannose pyrophosphate synthetase (GMPase), which catalyzes the generation of GDP-mannose, plays a very important role in the AsA biosynthesis in plants. The AsA content of *Arabidopsis* GMPase point mutant *vtc1-1* is only 25–30% of that in wild type plants [10]. Inhibiting the expression of GMPase genes can significantly reduce AsA content; in contrast, enhancing the expression of GMPase genes obviously increases AsA content in plants [10,11,12,13]. The AsA content in different tissues of potato was highly consistent with the transcript level of GMPase genes [11,13,14]. During the responses to stress, plants also can increase the AsA synthesis by regulating the expression of GMPase genes [10,14,15]. For example, light is one of the most important regulatory factors of AsA synthesis in plants, which not only activates the transcript of GMPase genes, but also suppresses the degradation of GMPase protein to regulate the AsA synthesis [15,16,17]. In rice, the expression of GMPase gene *OsVTC1-1* is regulated by light. Inhibiting the expression of *OsVTC1-1* significantly reduces the AsA synthesis induced by light [17]. These studies indicate that GMPase plays a key role in AsA synthesis.

AsA is widely involved in scavenging ROS in the responses of plants to various environmental stresses through direct or indirect pathways [10,18,19,20]. Under stress conditions, plants accumulate excess ROS, which can result in the peroxidation of plant lipids, proteins and other substances. The accumulated ROS disrupts the normal physiological activities in plants and causes serious damage to plants. The superoxide anion O_2_^−^ in plants can be converted into H_2_O_2_ by superoxide dismutase (SOD), and H_2_O_2_ can be further scavenged by AsA peroxidase (APX), so AsA is important for plants to scavenge ROS under stress conditions [10,14,19,20]. The studies from the analysis of salt tolerance of rice and tomato varieties showed that AsA content has a positive correlation with the salt tolerance of rice and tomato. The varieties with low AsA content were more sensitive to salt stress, while those with high AsA content were more tolerant to salt stress [21]. Plants can dynamically regulate endogenous AsA synthesis under stresses. Oxidative stresses, such as O_3_ and H_2_O_2_, can induce the expression of GMPase gene *VTC1* and promote AsA synthesis [10]. Under salt stress, the transcript factor AtERF98 regulates the expression of *VTC1* to enhance the synthesis of AsA and improves the salt stress tolerance of *Arabidopsis* [14]. Therefore, the GMPase gene has an important role in the regulation of AsA synthesis in the responses to environmental conditions [10,14,22,23].

The root is not only an important organ for plants to fix the plant body and absorb water and nutrients, but is involved in the responses of plants to environmental stresses. Under normal conditions, the root absorbs water and nutrients from the soil to support the growth and development of plants and maintain cellular homeostasis. Under unfavorable conditions, the root is forced to adopt several structural and cellular physiological functional modifications to adapt to adverse environmental conditions; even under certain conditions, such as high salt, the root is the front line to respond to environmental stress [24,25]. Therefore, though the responses of roots to abiotic stresses in plants are of particular importance [26,27], the root local abiotic stress responsive mechanism is still obscure. Rice encodes three GMPase homologous genes *OsVTC1-1*, *OsVTC1-3* and *OsVTC1-8*. A previous study showed that *OsVTC1-1* and *OsVTC1-3* were responsible for AsA synthesis in leaf and root, respectively. In contrast, *OsVTC1-8* may not be involved in AsA synthesis [17], and *OsVTC1-1* plays an important role in the response of rice to salt stress by regulating the redox homeostasis in the rice foliar organ [28]. The results from this study showed that rice GMPase gene *OsVTC1-3* plays a major role in scavenging the rapidly accumulated ROS in rice root and enhances the tolerance of rice to salt stress by regulating AsA synthesis in rice root.

## 2. Results

### 2.1. Salt Induces the Expression of OsVTC1-3 in Rice Root

GMPase plays an important role in AsA synthesis and stress responses in plants [10,19,20]. Previous studies showed that rice GMPase *OsVTC1-1* and *OsVTC1-3* were responsible for the foliar and root AsA synthesis, respectively [17], and the redox homeostasis in rice roots played an important role in the stress tolerance [29,30]. Is the regulated AsA biosynthesis by *OsVTC1-3* in rice roots involved the response of rice to environmental stresses, and what is role of *OsVTC1-3* in the response of rice to environmental stresses? To analyze the physiological function of *OsVTC1-3* in the response of rice to environmental stresses, firstly, we used real-time quantitative PCR (qPCR) to analyze the expression of *OsVTC1-3* in different rice tissues. The results showed that the expression of *OsVTC1-3* varied greatly in different tissues. The expression level of *OsVTC1-3* in rice root was very high, which was similar to that of *Actin*, and about more than five-times that in rice sheath and leaf. The expression level of *OsVTC1-3* in rice leaf and sheath was relatively low, which was about 18% of *Actin* (Figure S1). The promoter of *OsVTC1-3* was cloned, and the *GUS* reporter gene was used to further analyze the expression level of *OsVTC1-3* in different tissues of rice. The results also showed that *OsVTC1-3* was mainly expressed in root, and the expression level of *OsVTC1-3* in root was significantly higher than that in leaf and sheath (Figure S1). The above data were identical to the previous results [17].

**Figure 1:**
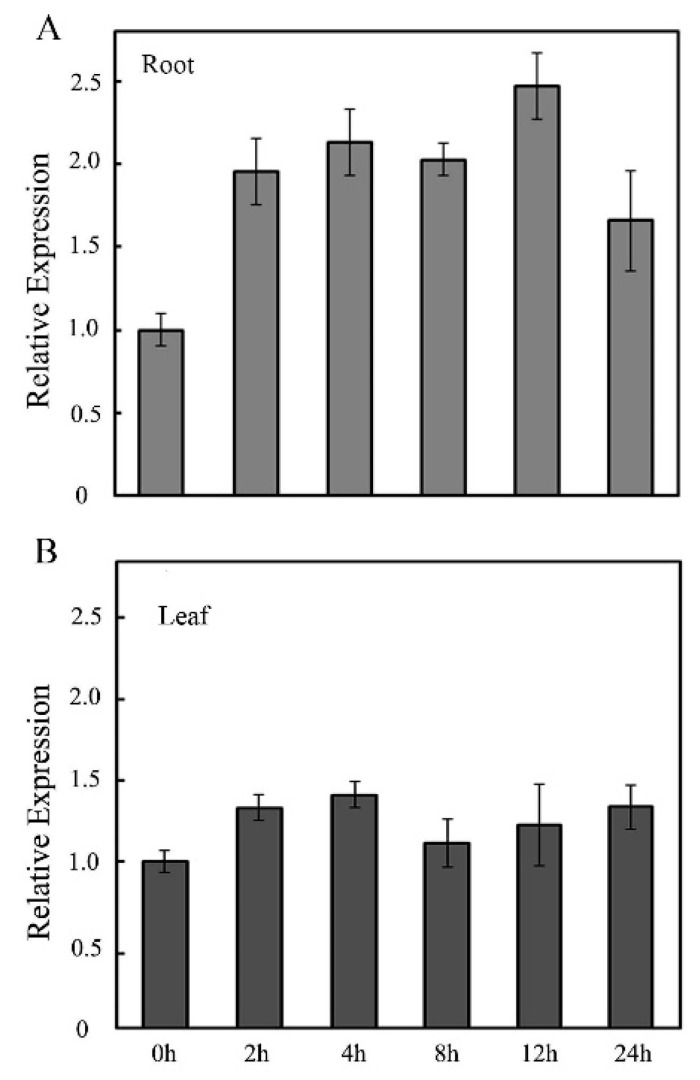
Salt induces *OsVTC1-3* expression in root. (**A**) The expression patterns of *OsVTC1-3* in rice roots under salt treatment were analyzed by qPCR. (**B**) The expression patterns of *OsVTC1-3* in rice leaves under salt treatment were analyzed by qPCR. After normalizing to internal control *Actin*, the transcript level of *OsVTC1-3* under control conditions (0 h) was assigned as “1”, and this figure shows the expression level of *OsVTC1-3* at other time point relative to 0 h. The experiments were repeated three times. The bars represent the SE (±) of three independent assays.

Studies showed that the expression of GMPase gene was closely related to the responses of plants to salt stress [20,21]. Salt stress is one of the main environmental stresses faced by rice and has a serious impact on rice yield [31,32]. The transcripts of *OsVTC1-3* had an important role in AsA biosynthesis of rice roots [17]. To study the role of *OsVTC1-3* in the response of rice to salt stress, the expression pattern of *OsVTC1-3* under salt treatment was analyzed. Salt treatment was performed on rice leaves and roots by spraying 150 mM NaCl solution and soaking in 150 mM NaCl solution for different time courses, respectively. The RNA was isolated, and the expression level of *OsVTC1-3* was analyzed by qPCR. The results showed that the expression level of *OsVTC1-3* in rice root with the 150 mM NaCl treatment at 2 h was about 1.8-times that under the control condition, and reached the highest at 12 h (Figure 1A). On the contrary, the expression level of *OsVTC1-3* in rice leaves with the 150 mM NaCl treatment did not show an obvious difference (Figure 1B), indicating that *OsVTC1-3* may be involved in the response of rice to salt stress in rice roots, but not leaves.

### 2.2. OsVTC1-3 Plays a Key Role in the Salt Stress Response of Rice

**Figure 2:**
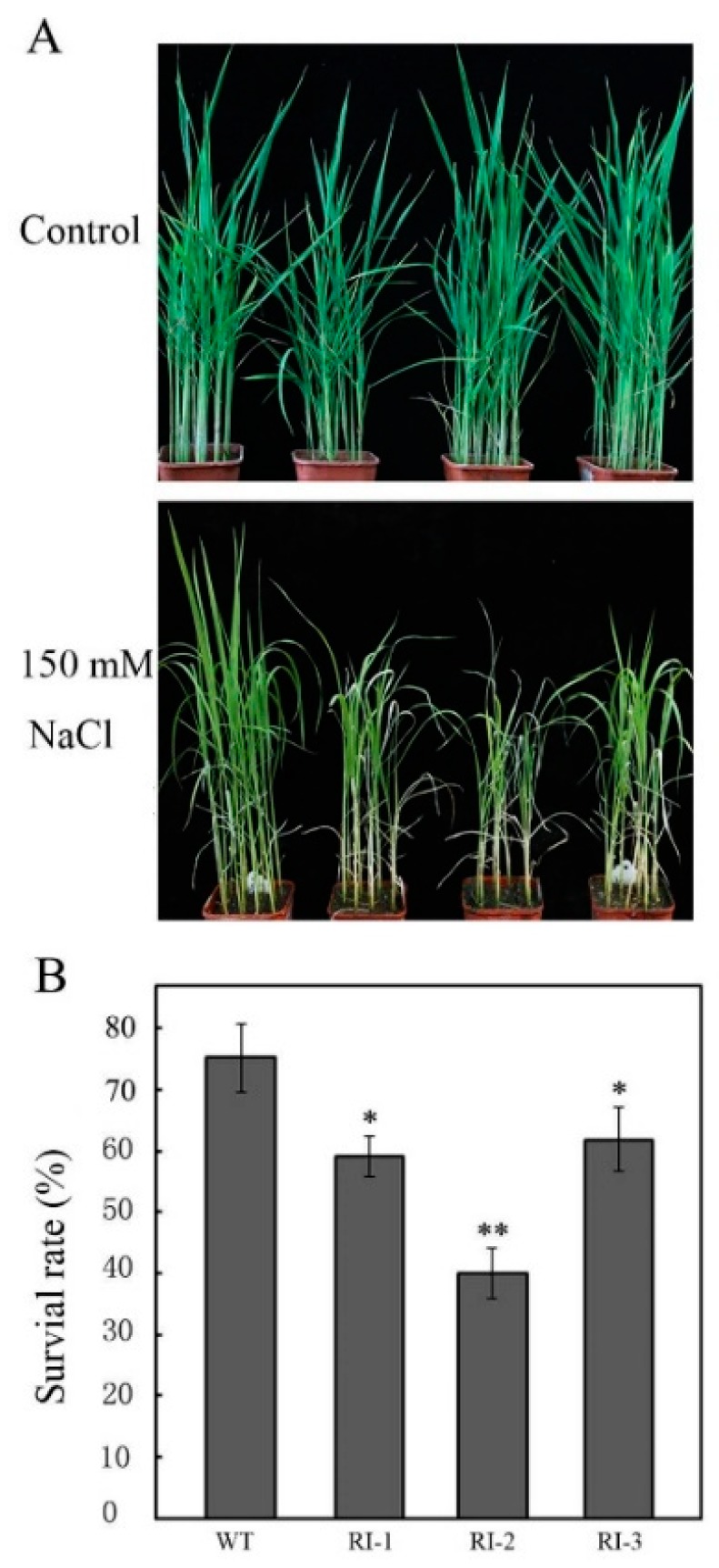
Inhibiting the expression of *OsVTC1-3* decreased the tolerance of rice to salt stress. (**A**) The phenotype of *OsVTC1-3* RNA interfering (RI) lines under salt stress. Control indicates that rice seedlings were grown under normal conditions, and NaCl indicates that seedlings were treated with 150 mM NaCl. (**B**) The survival rate percentage of *OsVTC1-3* RI lines after salt treatment in the experiment in (**A**). WT represents ZH17 rice variety; RI-1 and RI-2 indicate different independent RNA interference lines of *OsVTC1-3* in the ZH17 background, respectively. About 50–60 seedlings were used in each experiment. The bars represent SE (±) of three independent assays, and the asterisk indicates results significantly different from that of WT (** *p* < 0.01 and * *p* < 0.05). Significance was evaluated by using the *t*-test.

Previous studies showed that salt induced the expression of *OsVTC1-3* in rice roots, suggesting that *OsVTC1-3* may play an important role in the response of rice roots to salt stress. In order to analyze the function of *OsVTC1-3* in the salt tolerance of rice, we treated the high specificity *OsVTC1-3* RNAi lines, which only suppressed the expression of *OsVTC1-3* [17], with 150 mM NaCl. It was found that the salt tolerance of *OsVTC1-3* RNAi lines decreased significantly (Figure 2). After 10 days of salt treatment with another seven days of recovery, the survival rates of two-week-old *OsVTC1-3* RNAi seedlings were significantly lower than those of the wild type (WT). For example, the survival rate of RNAi line RNA interfering (RI)-2 was only about 40%; in contrast, the survival rate of WT was high, up to 75% (Figure 2B), after salt treatment. The above results showed that *OsVTC1-3* played a key role in the response of rice to salt stress.

### 2.3. Inhibiting the Expression of OsVTC1-3 Decreases the Ability of Rice Roots to Scavenge ROS under Salt Stress

**Figure 3:**
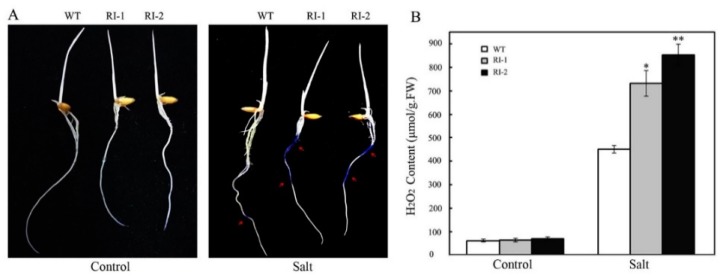
Inhibiting the expression of *OsVTC1-3* impairs the ability of rice roots to scavenge ROS under salt stress. (**A**) The O_2_^−^ content of *OsVTC1-3* RI roots under salt treatment. The rice seedlings were treated with 150 mM NaCl for 1 h and then were stained with NBT to show the content of O_2_^−^ in rice root. (**B**) The H_2_O_2_ content of *OsVTC1-3* RI lines under salt treatment. Control indicates that plants were grown under normal conditions, and NaCl indicates that plants were treated with 150 mM NaCl for five days. The arrow indicates the site where O_2_^−^ were stained with NBT and showed a blue deposit. About 40–50 plants were used in each experiment. Bars represent SE (±) of three independent assays, and the asterisk indicates that the results were significantly different from that of WT (** *p* < 0.01 and * *p* < 0.05). Significance was evaluated by the *t*-test.

The accumulated ROS under stress seriously affect the growth and development of plants, so the ability to scavenge ROS is of great significance for plants to improve the tolerance to stresses [33,34,35]. *OsVTC1-3* has an important role in AsA synthesis of rice roots, and AsA is closely related to scavenging of ROS. Therefore, we thought that *OsVTC1-3* may be involved in the response of rice to salt stress by regulating the ability of rice roots to scavenge ROS under salt stress. To analyze how *OsVTC1-3* regulates the salt stress response in rice, we analyzed the content of ROS in *OsVTC1-3* RNAi plants under salt stress. Rice roots were treated with 150 mM NaCl for 1 h and then were stained with the nitroblue tetrazolium (NBT) to analyze superoxide anion (O_2_^−^) content in rice roots after salt treatment. The results showed that O_2_^−^ accumulated rapidly in the roots of *OsVTC1-3* RNAi plants after salt treatment. In contrast, the O_2_^−^ content in WT roots was significantly lower than that in the root of *OsVTC1-3* RNAi plants (Figure 3A). Rice was treated with 150 mM NaCl for five days, and the contents of H_2_O_2_ were measured. The results showed that the H_2_O_2_ content was not significantly different in the roots of wild type and *OsVTC1-3* RNAi plants in the control condition, but after five days of salt treatment, an amount of H_2_O_2_ accumulated in the roots of *OsVTC1-3* RNAi plants. The H_2_O_2_ in the roots of *OsVTC1-3* RNAi plants was significantly higher than that in the root of wild type (WT). After five days of salt treatment, the H_2_O_2_ content increased about 12–15-times in the roots of different *OsVTC1-3* RNAi lines; in contrast, the H_2_O_2_ content in the roots of WT increased only six times (Figure 3B). The above results indicated that *OsVTC1-3* played an important role in controlling the ROS content in rice roots under salt stress.

### 2.4. AsA Plays an Important Role in OsVTC1-3 Regulating Salt Response in Rice Root

**Figure 4:**
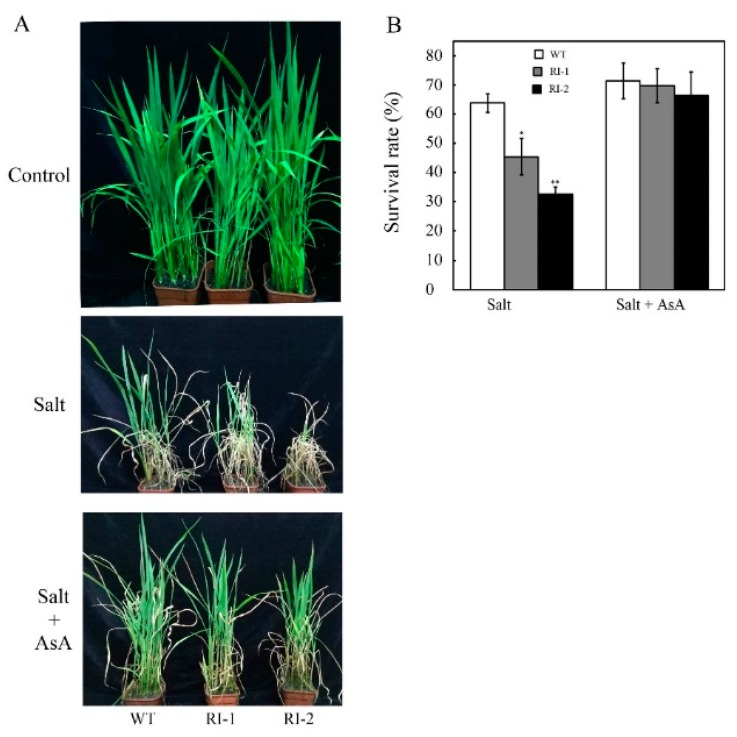
Exogenous ascorbic acid (AsA) rescues the tolerance of *OsVTC1-3* RI plants to salt stress. (**A**) Phenotype of *OsVTC1-3* RI plants grown in soil with or without supplying exogenous AsA under salt treatment. (**B**) The survival rate of *OsVTC1-1* RI plants with or without supplying exogenous AsA under salt treatment. Control indicates that rice seedlings were grown under normal conditions; NaCl indicates that rice seedlings were grown in soil by watering with 150 mM NaCl; AsA indicates that rice seedlings were grown in soil by supplied with 10 μM AsA; and NaCl + AsA represents rice seedlings grown in soil with 150 mM NaCl and 10 μM AsA. The above assays were repeated three times. About 50–60 seedlings were used in each experiment. The bars represent SE (±). The asterisk indicates results significantly different from WT (** *p* < 0.01 and * *p* < 0.05). Significance was evaluated by the *t*-test.

The data from above results showed that *OsVTC1-3* plays a key role in the salt-induced AsA synthesis in rice root under salt stress. It is well known that AsA has an important effect on scavenging the accumulating ROS under salt stress to enhance the tolerance of plants to salt stress, so we further analyzed the role of AsA in *OsVTC1-3* regulating the response of rice roots to salt stress by supplying exogenous AsA. The results showed that the salt tolerance of *OsVTC1-3* RNAi plants was mostly recovered after supplying 10 μM exogenous AsA (Figure 4). Similar to the above results, *OsVTC1-3* RNAi lines showed a salt-sensitive phenotype after 10 days of treatment with 150 mM NaCl; however, the exogenous AsA significantly enhanced the tolerance of *OsVTC1-3* RNAi lines to salt stress (Figure 4). The survival rates of *OsVTC1-3* RNAi lines RI-1 and RI-2 were about 46% and 33%, respectively. With exogenous AsA, the survival rate of *OsVTC1-3* RNAi lines RI-1 and RI-2 increased up to about 68% and 66%, respectively, which was almost the same as the 72% survival rate of the wild type (WT) (Figure 4B). This result indicated that AsA plays a key role in *OsVTC1-3* regulating the response of rice roots to salt stress.

### 2.5. OsVTC1-3 Regulates AsA Synthesis in Rice Root under Salt Stress

**Figure 5:**
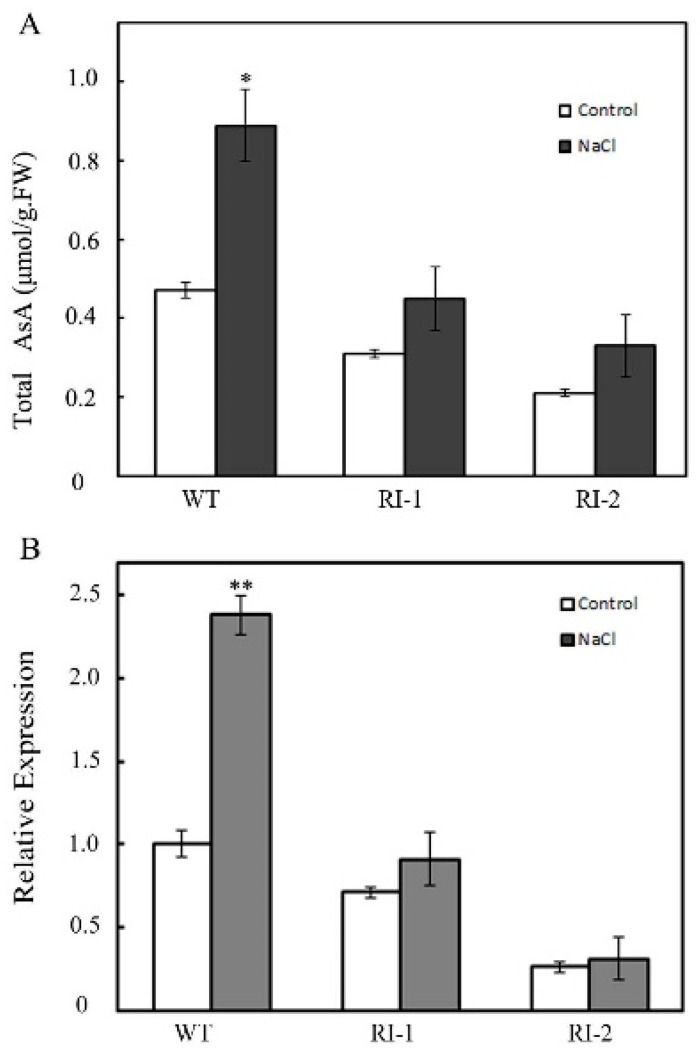
*OsVTC1-3* regulates AsA synthesis in rice root under salt stress. (**A**) The transcript level of *OsVTC1-3* in WT and *OsVTC1-3* RI plants under salt stress. Control indicates that plants were grown under normal conditions, and NaCl indicates that plants were treated with 150 mM NaCl for 12 h. (**B**) The AsA content of *OsVTC1-3* RI plants under salt stress. Control indicates that plants were grown under normal conditions, and NaCl indicates that plants were treated with 150 mM NaCl for five days. The bars represent SE (±) of three independent assays, and the asterisk indicates results significantly different from that of the normal condition (** *p* < 0.01 and * *p* < 0.05). Significance was evaluated by the *t*-test.

Previous studies have shown that *OsVTC1-3* had an important regulatory role in AsA synthesis in rice roots [17]. What is the relationship between *OsVTC1-3* regulating AsA synthesis and salt stress in rice? Firstly, we analyzed the effects of salt stress on AsA synthesis in rice. Rice was treated with 150 mM NaCl for five days, and then, the AsA content in rice roots was measured. The results showed that the AsA content in the roots of *OsVTC1-3* RNAi plants was less than that of WT under salt treatment. For example, the AsA content in RI-2 roots increased only about 0.11 μM/g·FW (from 0.21 μM/g·FW–0.32 μM/g·FW) after five days of salt treatment; in contrast, the AsA content in WT roots increased 0.42 μM/g·FW (from 0.47 μM/g·FW–0.89 μM/g·FW). The increasing AsA content in WT roots was about four-times that in RI-2 (Figure 5A). Further studies found that the expression of *OsVTC1-3* in the *OsVTC1-3* RNAi plants was inhibited under salt stress. The expression of *OsVTC1-3* in WT under salt was about 2.4-times as high as that under the normal growing conditions; in contrast, there were no significant differences in *OsVTC1-3* RNAi roots between salt treatment and normal growing conditions (Figure 5B). The above results indicated that salt-induced expression of *OsVTC1-3* was necessary for AsA synthesis in rice roots under salt stress.

## 3. Discussion

Root is an important organ for plants to absorb water and nutrients, as well as the responses to abiotic stresses, such as salt and drought [23]. Salt is one of the most important adverse environmental factors that seriously impairs rice production. However, few studies have focused on the mechanisms of plant roots in salt stress response [36]. This study found that the expression of GMPase gene *OsVTC1-3* in rice roots under salt stress is a key for rice to accelerate AsA synthesis to scavenge the accumulated ROS and enhance the salt tolerance.

GMPase is a key enzyme of the AsA synthesis pathway in higher plants. The activity of GMPase has an important effect on the AsA synthesis of plants [3,5,6]. Plants can regulate the activity of GMPases in vivo by regulating their transcript or protein stability [14,16,17,23]. For example, salt stress and light can induce the expression of the GMPase gene to promote AsA synthesis [14,15,17]. The expression of *OsVTC1-3* was predominant in rice roots. When the function of *OsVTC1-3* was impaired in *OsVTC1-3* RI plants, the AsA content in rice roots decreased obviously, but the AsA content in leaves did not change significantly, indicating that the transcript level of *OsVTC1-3* was closely related to the AsA synthesis of rice root (Figures S1 and S2). The expression of *OsVTC1-3* was induced by salt stress. The inhibition of *OsVTC1-3* expression induced by salt in *OsVTC1-3* RI plants significantly reduced the AsA synthesis in rice roots (Figure 5), indicating that the transcript of *OsVTC1-3* was important for rice root to improve AsA synthesis under salt stress. Different from *Arabidopsis*, which has only one GMPase gene, rice has three homologous genes encoding full-length GMPase, so rice can specifically regulate GMPase genes to express in different tissues and organs to maintain AsA synthesis in the response to environmental stresses.

The accumulated ROS in plants has an important influence on plant growth and development in adversity. Therefore, the ability of plant organs and tissues to scavenge ROS is closely related to the adaptability of plants to adverse environments [18,19,20,22]. Studies have shown that GMPase plays an important role in scavenging ROS in leaves of *Arabidopsis* and rice [10,22,37]. The mutation of the GMPase gene reduced the ROS scavenging ability of *Arabidopsis*, which not only significantly impaired the tolerance of *Arabidopsis* to O_3_ and other oxidizing substances, but also decreased the tolerance of *Arabidopsis* to salt stress [10,22]. Inhibiting the expression of *OsVTC1-3* resulted in the rapid accumulation of H_2_O_2_ in rice roots under salt stress; in contrast, there was no obvious H_2_O_2_ accumulation in rice leaves (Figure 3). Different from *OsVTC1-3*, the previous study showed that rice GMPase gene *OsVTC1-1* played an important role in scavenging ROS in rice leaves [28], indicating that rice GMPase homologous genes *OsVTC1-1* and *OsVTC1-3* may be involved in the salt response in rice different organs, *OsVTC1-1* in leaves and *OsVTC1-3* in roots by regulating ROS dynamics.

Under stress conditions, plants can regulate the expression of GMPase genes to upregulate AsA synthesis and enhance the stress tolerance of plants [14,23,35]. For example, the expression of *VTC1* and other AsA synthesis genes was activated by transcription factor AtERF98 in *Arabidopsis* to promote AsA synthesis and enhance the salt tolerance of *Arabidopsis* [14]. The exogenous AsA rescued most tolerance of *OsVTC1-3* RI plants to salt stress, showing that the role of *OsVTC1-3* in regulating AsA synthesis was important for rice roots to scavenge ROS and enhance the tolerance of rice to salt stress (Figure 4) Salt stress rapidly induced *OsVTC1-3* expression and increased AsA biosynthesis in rice roots (Figure 1), and decreasing the expression of *OsVTC1-3* significantly impaired the salt-induced AsA synthesis in rice roots (Figure 5), indicating that *OsVTC1-3* is involved in rice salt response by regulating AsA synthesis in rice roots under salt stress. In contrast, the homologous *OsVTC1-1* played an important role in salt response in rice leaves due to regulating AsA biosynthesis in rice leaves [17,28]. In all, the results from the present work suggested that, different from homologous *OsVTC1-1*, which regulates AsA biosynthesis and the salt response of rice leaves, rice can specifically activate the transcript of *OsVTC1-3* in rice roots to promote AsA synthesis to scavenge ROS in rice roots to improve the salt tolerance of rice under salt stress.

## 4. Materials and Methods

### 4.1. The Cultivation of Plant Materials

The wild type rice variety used in this work is (*Oryza sativa* L. ssp. *japonica* cv. Zhonghua 17) (ZH17). *OsVTC1-3*’s RNA interfering materials (RI-1 and RI-2) were two independent transgenic lines with the knocked down expression of *OsVTC1-3* by RNA interfering technology in the background of ZH17. The expression of *OsVTC1-3* in RI-1 and RI-2 materials was approximately 60% and 20% of that in the wild type, respectively (the expression of *OsVTC1-3* in RI-1 and RI-2 was shown by Qin et al. [17].

To germinate rice seeds, the rice seeds were soaked and cultured at 37 °C for 2 days. To analyze the expression of *OsVTC1-3* with the GUS reporter gene, the germinated rice seeds were grown in the liquid of 1/2 MS (Murashige and Skoog). To grow rice in soil, the germinated seeds were planted in soil and then grown in a greenhouse at 25–30 °C with a 16-h light and 8-h dark cycle.

### 4.2. The Generation of Transgenic Rice

To generate *OsVTC1-3* RNA interference (RNAi) plants, the specific 3′-UTR sequence of *OsVTC1-3* was designed as the targeted sequence. The specific targeted sequences were cloned into the pUCCRNAi vector using the *Xho* I and *Bgl* II sites, and then, the constructed pUCCRNAi vector was digested with *Sal* I and *BamH* I. Following this, the digested DNA fragment was linked with the digested fragment of *Xho* I and *Bgl* II to get the DNA fragments that contained the forward and reverse targeted sequences, which further were cloned into the plant vector pCAMBIA2300 by the *Pst* I site. Then, the resultant plasmid was introduced into ZH17 by using Agrobacterium-mediated transformation. The transformed plants were selected by G418. The efficiency and specificity of the RNAi lines were confirmed by real-time quantitative PCR (Q-PCR). Transgenic rice lines with reduced expression levels of *OsVTC1-3* were denoted as RI-x (here, x indicates the numbering of different independent transgenic lines). The primers for RNAi vector construction were positive primer 5′-CTCTCGAGCCTCCTTTTATGTTATGGTA-3′ and reverse primer 5′-CCAGATCTAAGAACAAAGTACAAGGCTG-3′. The primers for identifying the expression level of *OsVTC1-3* in RNAi lines were positive primer 5′-CGAGGGACTACATCACCGGG-3′ and reverse primer 5′-CTCGTGGACGAGCACGTTG-3′.

### 4.3. The Analysis of OsVTC1-3 Expression by qPCR

A 0.2-g sample was used to extract total RNA by TRIzol (No. DP421, Tian Gen, Beijing, China). Then, cDNA was synthesized according to the kit (No. 18080051, Invitrogen, Carlsbad, CA, USA). The 1 μL of diluted cDNA (the Ct value of the *Actin* of the cDNA template was about 20) was used as the template. Then, the qPCR was carried out in accordance with the experimental manual of IQ5 (BIO-RAD, Hercules, CA, USA) to detect the relative expression level of *OsVTC1-3*, and the detail was described by Qin et al. [17]. Actin was used as the internal control. Each experiment was repeated 3 times, and the results showed the means and experimental errors of three independent experiments. The qPCR primers for *OsVTC1-3* were positive primer 5′-CGAGGGACTACATCACCGGG-3′ and reverse primer 5′-CTCGTGGACGAGCACGTTG-3′, and qPCR primers for *Actin* were positive primer 5′-GACCTTGCTGGGCGTGAT-3′ and reverse primer 5′-GTCATAGTCCAGGGCGATGT-3′.

### 4.4. The Determination of the Content of AsA

Zero-point-one-seven-five grams of ascorbic acid were added into a 15-mL tube and 10 mL 6% perchloric acid (HClO_4_) added to prepare the 100 mM AsA solution. The AsA mother liquor was diluted with 6% perchloric acid to 10 μM, 5 μM, 1 μM, 500 nM, 200 nM, 100 Nm and 50 nM AsA standard solutions. Then, each 200-μL standard sample was added into a 2-mL centrifuge tube with 1800 μL 0.2 M sodium butyrate buffer (pH = 12.7) and set on ice in the dark for 20 min. Following that, the absorption value of each sample was measured at A_265_ to make the standard curve.

The measurement protocol was described by Zhang et al. [14]. About 0.2-g rice samples were ground into a fine power in liquid nitrogen, and then, the ground well samples were transferred into 5-mL centrifuge tubes containing 1 mL 6% HClO_4_, then on ice for 5 min, avoiding light. Following that, samples were centrifuged at 12,000× *g* rpm for 10 min. Then, 200 μL of supernatant were taken into a 5-mL centrifuge tube containing 1800 μL 0.2 M sodium succinate buffer (pH = 12.7) and 60 μL 1 M DTT. After being mixed and left at room temperature in the dark for 20 min, the OD1 was measured at A_265_ by a spectrophotometer. Another 200 μL supernatant were added into tube containing 1800 μL, 0.2 M sodium succinate buffer (pH = 12.7) and 4U ascorbic acid oxidase (AAO, No. PM0131, Sigma, Tokyo, Japan). After being mixed and left at room temperature in the dark for 20 min, the OD2 was measured at A_265_. The total AsA content of each sample was calculated by the value of OD1–OD2 according to the standard curve.

### 4.5. The Identification of the Salt Tolerance of Rice Materials

The germinated rice seeds were planted and grown in pots for 2 weeks and then treated with 150 mM NaCl solution or 150 mM NaCl and 10 μM AsA solution. After 5–10 days of salt treatment, rice seedlings were restored under normal growing conditions for another 7 days. Then, photos were taken of different rice materials, and their survival rates were counted.

### 4.6. The Determination of O_2_^−^ Content in Rice by NBT Staining

The 2-week rice seedlings were treated with 150 mM NaCl for 1 h, and then, the O_2_^−^ content in rice roots was detected by NBT staining. The rice roots treated with water (control) and salt (NaCl) were placed in the tubes with 10 mL of staining solution (including 1 mg/mL DAB; 50 mM, pH = 3.8, NaAc-HAc), respectively. After being evacuated by vacuum for 10 min, these samples were placed in darkness at room temperature for 8 h. The roots were taken out of tubes and then rinsed with distilled water, before the samples were decolored by 10 mL 95% ethanol solution overnight. Then, the dyeing samples were observed and pictures of them taken [14].

### 4.7. The Determination of H_2_O_2_ Content

Rice seedlings were treated with salt for 5 days and then were used to measure the H_2_O_2_ content in rice root. About 0.1 g of rice root were ground into a fine power in liquid nitrogen, then the ground well samples were moved into tubes containing 1 mL of precooled acetone, on an ice bath for 3 min. Following that, H_2_O_2_ was extracted from the samples, and the H_2_O_2_ content was determined according to the H_2_O_2_ measuring kit (No. S0051, Bi Yun Tian, Shanghai, China). The detailed process was also described by the protocol of the kit.

### 4.8. GUS Staining of Rice Tissue

The roots, leaves and sheaths of 10-day water-cultured rice seedlings were taken into 10-mL tubes containing 5 mL of GUS staining solution, respectively. The rice materials in GUS-dyed solution were evacuated for 15 min with a vacuum pump and then incubated at 37 °C for 24 h. Following that, the chlorophyll of rice materials was removed by ethanol, and then, the expression level of the *GUS* reporter gene in the rice tissue was observed. The detailed process was described in the GUS Staining Kit (No. G3060, Solarbio, Beijing, China).

## Supplementary Materials

Supplementary Materials can be found at http://www.mdpi.com/1422-0067/19/11/3347/s1.
