# Supplementary material for: The Synthesis of Ascorbic Acid in Rice Roots Plays an Important Role in the Salt Tolerance of Rice by Scavenging ROS
Source: Int J Mol Sci. 2018 Oct 26;19(11):3347. doi: 10.3390/ijms19113347 (PMC6275051; doi:10.3390/ijms19113347)
Supplement: Supplementary file 1 [file ijms-19-03347-s001.zip › supplementary/supplementary figure legend.docx]

**Supplementary Figure Legend**

**Figure S1** **The expression of *OsVTC1-3* is predominant in rice root.**

(A) The expression of *OsVTC1-3* in rice root, sheath and leaf was analyzed by qPCR. *Actin* was used as internal control, and the transcript level of *Actin* was assigned as “1”. This figure shows the expression level of *OsVTC1-3* was relative to *Actin*. The experiments were repeated three times. The bars represent SE (±) of three independent assays. (B) The expression of *OsVTC1-3* in rice root, sheath and leaf was analyzed by report gene *GUS*.

**Figure S2** **The AsA content in the leaf, sheath and root of *OsVTC1-3* RI plants.** (A) The AsA content in the leaf of *OsVTC1-3* RI plants. (B) The AsA content in the sheath of *OsVTC1-3* RI plants. (C) The AsA content in the root of *OsVTC1-3* RI plants. The bars represent SE (±). The asterisk indicates results significantly different from WT (**P＜0.01 and *P＜0.05). Significance was evaluated using the *t*-test.
