# Supplementary figures and images for: The Synthesis of Ascorbic Acid in Rice Roots Plays an Important Role in the Salt Tolerance of Rice by Scavenging ROS
Source: Int J Mol Sci. 2018 Oct 26;19(11):3347. doi: 10.3390/ijms19113347 (PMC6275051; doi:10.3390/ijms19113347)

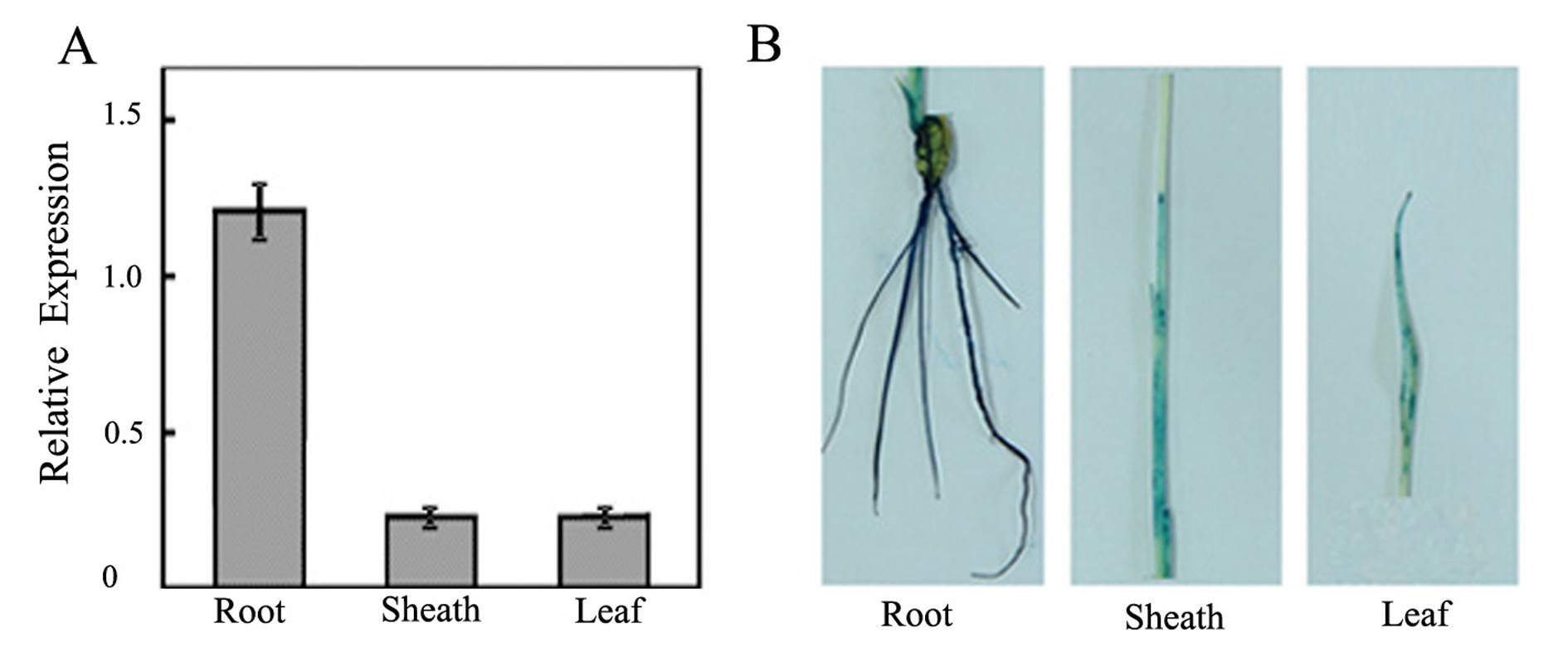

Supplement: Supplementary file 1 [file ijms-19-03347-s001.zip › supplementary/Figure S1.jpg]

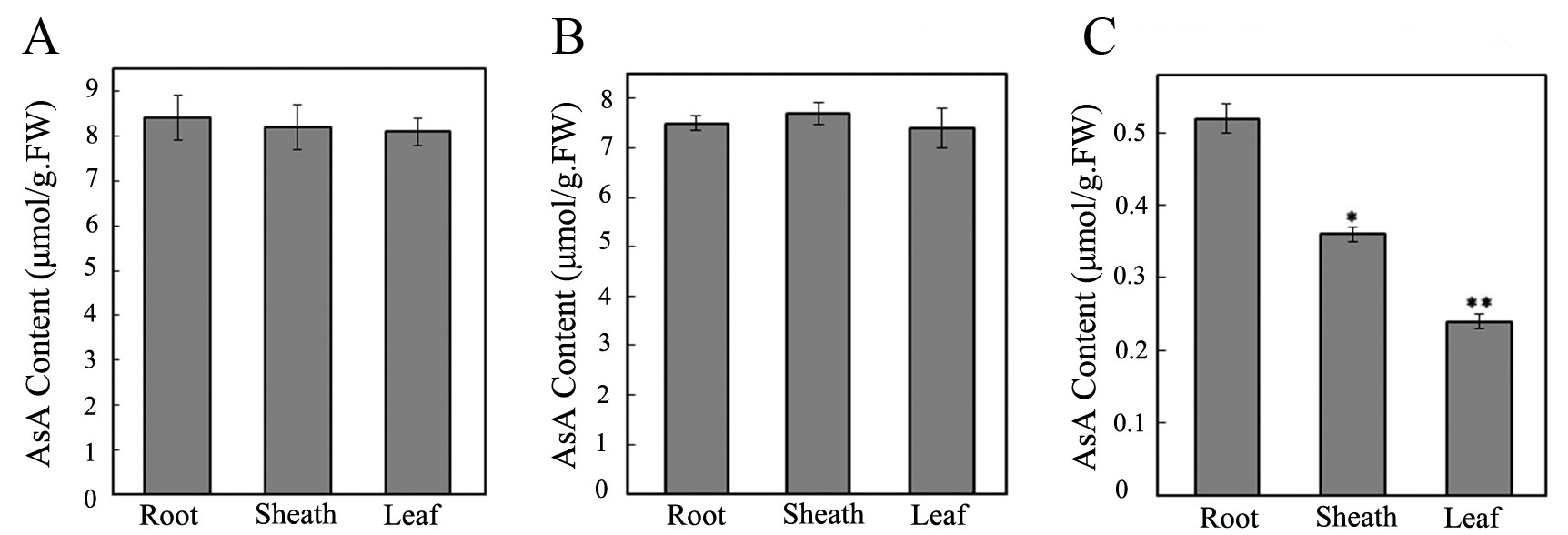

Supplement: Supplementary file 1 [file ijms-19-03347-s001.zip › supplementary/Figure S2.jpg]
